# Double jeopardy: how *BRCA2*, *TP53*, and *DNMT3A* redefine the link between infertility and cancer risk

**DOI:** 10.3389/fcell.2026.1803566

**Published:** 2026-06-02

**Authors:** Zhengbin Huang, Musavir Abbas, Ansar Hussain, Ming Long, Muhammad Muzammal, Kaiyong Song

**Affiliations:** 1 School of Business Administration, Chongqing Technology and Business University, Chongqing, China; 2 Centre for Reproduction and Genetics, First Affiliated Hospital of USTC, Hefei National Laboratory for Physical Sciences at Microscale, School of Basic Medical Sciences, Biomedical Sciences and Health Laboratory of Anhui Province, Institute of Health and Medicine, Hefei Comprehensive National Science Centre, Division of Life Sciences and Medicine, University of Science and Technology of China, Hefei, China; 3 Chongqing Precision Medical Industry Technology Research Institute, Chongqing, China; 4 Gomal Centre of Biochemistry and Biotechnology, Gomal University, Dera Ismail Khan, Pakistan; 5 Nephrology Department of Chongqing Liangjiang New Area Traditional Chinese Medicine Hospital Chongqing, Chongqing, China

**Keywords:** *BRCA2*, genomic instability, homologous recombination, oncofertility, PGT-M, premature ovarian insufficiency, *TP53*

## Abstract

**Background:**

Germline mutations in genes governing DNA repair, cell cycle regulation, and epigenetic modification are now recognized as common etiological factors for both cancer predisposition and reproductive dysfunction. This reveals a profound intersection between reproductive biology and oncogenesis.

**Method:**

A systematic narrative review was conducted. The literature search spanned PubMed/MEDLINE, Scopus, and Web of Science using keywords and MeSH terms related to infertility phenotypes, cancer predisposition syndromes, and shared molecular mechanisms (e.g., DNA repair, epigenetics).

**Outcomes:**

The analysis identifies a core set of genes including *BRCA1/2, TP53, ATM*, and *DNMT3A* with pleiotropic roles. Mechanistically, defects in pathways like homologous recombination and mismatch repair disrupt meiotic fidelity, causing gametogenesis failure (e.g., *BRCA2*-mediated azoospermia), while fostering genomic instability that drives carcinogenesis. Clinically, distinct phenotypes emerge, such as *BRCA1*-associated premature ovarian insufficiency and *TP53*-related germ cell apoptosis. Mouse models validate these links, showing that homozygous loss often causes complete sterility. Translational strategies, including PGT-M for high-risk variants and microsurgical testicular sperm extraction (micro-TESE) with intracytoplasmic sperm injection (ICSI) for azoospermia, offer concrete methods for risk mitigation within integrated oncofertility programs.

**Conclusion:**

Infertility and cancer susceptibility are fundamentally linked through shared genetic vulnerabilities and molecular pathways. This necessitates a paradigm shift toward dual-risk management, involving universal genetic screening in idiopathic infertility, the development of polygenic risk models, and close multidisciplinary collaboration. While ethical challenges persist, these advances pave the way for personalized care that simultaneously addresses reproductive and oncologic health.

## 1 Introduction

Infertility remains a significant global health challenge, affecting approximately 15% of couples, with 10%–30% of cases attributed to underlying genetic factors (Obeagu and Infertility, 2023). A growing body of evidence highlights an unexpected convergence between infertility and cancer predisposition, where pathogenic variants in genes traditionally associated with oncogenesis also disrupt reproductive function (Tiwari et al., 2024). This intersection suggests that shared molecular pathways govern both gametogenesis and somatic cell stability, with defects manifesting as reproductive failure and increased cancer risk (Fang et al., 2022). Carriers of germline mutations in high-penetrance cancer risk genes such as *BRCA1*, and *BRCA2* frequently exhibit subfertility, ranging from premature ovarian insufficiency to azoospermia (Barili et al., 2024; Panier and Wang, 2024). The mechanistic links between infertility and cancer susceptibility often center on DNA damage response pathways (Xu et al., 2024). Genes involved in homologous recombination repair, including *BRCA1, BRCA2,* and *PALB2,* are critical for resolving DNA double-strand breaks (DSBs) during meiosis (Lingg and Rottenberg SFrancica, 2022). In their absence, gametogenesis is disrupted, leading to meiotic arrest, apoptosis of developing germ cells, and subsequent infertility (García-Rodríguez et al., 2018). Notably, these same genes are essential for suppressing tumorigenesis in proliferating somatic cells, where their dysfunction results in genomic instability and malignant transformation (Gorodetska and Kozeretska IDubrovska, 2019). Similarly, cell cycle regulators such as *TP53* and *ATM* play pivotal roles in monitoring DNA damage in both germ and somatic cells (Timmerman et al., 2021). Mutations in these genes can trigger germ cell apoptosis, leading to gonadal failure, while simultaneously permitting unchecked somatic proliferation, a hallmark of cancer.

Beyond DNA repair, epigenetic dysregulation further bridges infertility and cancer risk (Dutta et al., 2024). Genes encoding chromatin modifiers, including *DNMT3A* and *MTHFR*, influence both gametogenesis and oncogenesis through alterations in DNA methylation and histone modification (Rotondo et al., 2021). For example, aberrant methylation patterns in sperm DNA have been linked to oligospermia, while similar epigenetic disruptions in somatic cells can activate oncogenes or silence tumor suppressors (Zhang et al., 2024a). Hormonal pathways also contribute to this overlap, as illustrated by *PTEN* mutations, which perturb folliculogenesis and endometrial function while predisposing to breast and thyroid cancers (Denaro et al., 2023).

Clinically, these shared vulnerabilities necessitate a paradigm shift toward integrated oncofertility care. Genetic screening for cancer risk variants in infertile populations could identify individuals at heightened risk for malignancies, enabling early surveillance and intervention (Evans OManchanda and Manchanda, 2020). Conversely, cancer patients harboring these mutations should be counseled on potential fertility impairments and offered preservation options prior to gonadotoxic therapies (Medicine, 2018; Parikh et al., 2023).

This review addresses three critical gaps:

1. Mechanistic: How do DNA repair, epigenetic, and hormonal disruptions simultaneously impair fertility and promote cancer?
2. Clinical: What is the evidence-based phenotypes (*BRCA1 POI, ATM* oligospermia) that warrant genetic testing?
3. Translational: How can assisted reproductive technologies (PGT-M, ICSI) and oncofertility programs mitigate risks?

## 2 Methodology

### 2.1 Literature search strategy

The systematic literature search involved PubMed/MEDLINE, Scopus, and Web of Science, and the literature published. The search was limited using keywords and MeSH terms that covered three main concepts underlying the search: (1) infertility and reproductive phenotypes (e.g., “premature ovarian insufficiency,” “(POI),” “diminished ovarian reserve”, “(DOR),” and “azoospermia,” “gametogenesis”); (2) cancer predisposition (e.g., “hereditary cancer syndrome,” “*BRCA1*,” “*TP53*,” “Lynch syndrome); and (3) common mechanisms (e.g., “DNA repair,” “homologous recombination,” “epigenetics, “oncofertility”. The connection between these notions was expressed using logical operators (AND, OR). Manual screening of the reference lists of the retrieved articles was also done to find more relevant publications.

### 2.2 Study selection and data extraction

The title and abstract filters were used to filter articles with the full-text review being conducted to filter out ineligible articles. The studies that were selected had to explore the role of a cancer predisposition gene in a reproductive phenotype, clarify a common mechanism of action between oncogenesis and gametogenesis, or provided information on clinical interventions such as fertility preservation or PGT-M in carriers of mutations. Information of chosen sources was pulled out in a standardized format, including gene(s), type of study, mechanistic information, clinical phenotypes and translational applications.

### 2.3 Data synthesis

Since the narrative review design was used, a formal meta-analysis was not implemented. Rather, synthesis of evidence has been done thematically to fulfill objectives of the review: the clarification of common mechanistic pathways, clinical phenotype, and translation implications. Our evidence selection criteria were based on high quality evidence obtained in functional research and clinical cohorts, with clearly differentiating between established and emerging associations in the text, tables and figures.

## 3 Shared genetic vulnerabilities linking gametogenesis failure and cancer risk

### 3.1 Shared genetic vulnerabilities: cancer predisposition and reproductive dysfunction

The growing recognition of genes with dual roles in cancer predisposition and infertility reveals fundamental connections between reproductive biology and oncogenesis (Tarín et al., 2015). Among DNA repair genes, *BRCA1* and *BRCA2* stand out for their critical functions in both maintaining genomic stability and ensuring proper gametogenesis (Dias Nunes and Demeestere IDevos, 2023). *BRCA1* mutations are associated with premature ovarian insufficiency through diminished ovarian reserve, as evidenced by lower anti-Müllerian hormone (AMH) levels and reduced mature oocyte yield. Oktay et al. (2010) correlated *BRCA1* with occult primary ovarian insufficiency, while Derks-Smeets et al. (2017) showed that *BRCA1* mutation carriers produced lower numbers of mature oocytes compared to controls. Turan et al. (2018) reported lower oocyte yield in *BRCA2*-mutated patients, and Lambertini et al. (2018) found that *BRCA*-positive women tend to recover fewer oocytes than *BRCA*-negative breast cancer patients. Beyond heterozygous carriers, biallelic *BRCA1* frameshift mutations (c.470_471del; c.791_794del) were recently identified in a patient with isolated diminished ovarian reserve without Fanconi anemia, demonstrating residual DNA repair activity via a truncated del11q isoform (Helbling-Leclerc et al., 2024). Regarding male fertility, the polymorphism N372H in *BRCA2* has been associated with idiopathic male infertility with azoospermia or severe oligozoospermia (OR = 1.49, 95% CI 1.06–1.97, P = 0.02) (Zhoucun et al., 2006).

Similar dual phenotypes appear in other homologous recombination genes like *PALB2, BRIP1,* and *RAD51C*, which all contribute to both cancer risk and impaired oocyte development (Toh and Ngeow, 2021; Hanson et al., 2023; Wang D. et al., 2024; Pavanello et al., 2020).

The Fanconi anemia pathway genes (*FANCA, FANCC, FANCM*) demonstrate how DNA repair defects can simultaneously cause germ cell depletion and hematologic malignancies (Nalepa and Clapp, 2018). Biallelic *FANCM* mutations were first identified in a Finnish family with premature ovarian insufficiency (Kotsakis et al., 2014; Fouquet et al., 2017) and later confirmed in a large cohort of POI patients (Heddar et al., 2022), establishing *FANCM* as a cause of female infertility. Specifically, bi-allelic recessive loss-of-function variants in *FANCM* have been convincingly demonstrated to cause azoospermia and Sertoli cell-only syndrome (SCOS), as shown by Kasak et al. (Kasak et al., 2018) in Estonian brothers with compound heterozygous *FANCM* variants (p.Gln498Thrfs∗7 and c.4387−10A>G). Two additional NOA-affected case subjects with independent *FANCM* homozygous nonsense variants (p.Gln1701∗ and p.Arg1931∗) were identified, establishing *FANCM* as a definitive cause of male infertility. *ATM* and *CHEK1* mutations disrupt both fertility and cancer protection. *ATM* defects cause spermatogenic arrest and leukemia risk, while *CHEK1* variants have been associated with premature ovarian failure and breast cancer susceptibility in some studies (Sadeghi, 2023; Guo et al., 2025). These dual effects stem from impaired DNA damage response - triggering germ cell loss while permitting somatic cell survival*.* Clinically, this requires combined fertility and cancer monitoring for mutation carriers (Wang Y. et al., 2023). Mismatch repair genes like *MLH1, MSH2*, and *MSH6*, known for their association with Lynch syndrome cancers, also play essential roles in meiotic recombination, with their dysfunction leading to gamete aneuploidy and infertility including azoospermia and oligozoospermia (Tamura et al., 2019).

Cell cycle regulators show equally important dual functions. *TP53* mutations trigger germ cell apoptosis causing gonadal failure while predisposing to multiple cancers in Li-Fraumeni syndrome (Rocca et al., 2022). *PTEN*, a critical tumor suppressor in breast and thyroid cancers, also regulates ovarian function and endometrial receptivity (Ren BZhu and Zhu, 2022). The *STK11* gene, mutated in Peutz-Jeghers syndrome, causes both gonadal tumors and gastrointestinal malignancies. Even metabolic genes like *FH* demonstrate this pattern, with mutations affecting both implantation and renal cancer development (Bennett et al., 2021).

Several genes show tissue-specific effects that highlight the complexity of these dual roles. *DNMT3A* mutations drive leukemogenesis through global hypomethylation while causing male infertility via localized hypermethylation at imprinted loci (Rotondo et al., 2021). The *DKC1* gene’s role in telomere maintenance means its dysfunction accelerates both ovarian aging and epithelial carcinogenesis.

### 3.2 Integrating preclinical and clinical evidence: from mouse models to human disease

Germline cancer mutations impair fertility through distinct mechanisms. Our analysis of 81 genes (Table 1) shows DNA repair genes (*BRCA1/2, PALB2, ATM, FANCM*) most strongly affect both cancer risk and fertility. To clearly delineate experimental evidence from clinical correlations, this section is organized into three parts: (i) preclinical evidence from mouse models, (ii) clinical evidence in humans, and (iii) integrated mechanistic synthesis.

**TABLE 1:** Germline cancer predisposition genes and associated reproductive phenotypes.

Sr No	Gene	Primary associated Cancer(s)	Known reproductive/Infertility role	Variant type/Mutation type	References
1	*APC*	Colorectal, breast, testicular yolk sac tumor	Sertoli cell junction disruption; BTB dysfunction; germ cell loss; testicular yolk sac tumor (infantile)	Promoter hypermethylation (70% of infantile YSTs); LOH at 5q21 (30% of YSTs); truncating mutations in MCR region (colorectal cancer)	Tanwar and Zhang LTeixeira (2011); Kato et al. (2006); Okpanyi et al. (2011); Fearnhead and Britton MPBodmer (2001); Aoki KTaketo and Taketo (2007)
2	*ATM*	Breast, lymphoma, leukemia	Nonobstructive azoospermia (NOA); spermatogenic arrest; testicular atrophy	Promoter variant: c.-111G>A (rs189037); missense: c.8565T>G (p.Ser2855Arg); c.3161C>G (p.Pro1054Arg); c.4258C>T (p.Leu1420Phe)	Cioppi and Rosta VKrausz (2021); Takubo et al. (2006); Gao et al. (2010); Li et al. (2013)
3	*ATR*	Breast, ovarian	Meiotic arrest in gametes; Seckel syndrome with infertility	Biallelic loss-of-function: c.7636-2A>G (splice-site); c.6902G>A (p.Arg2301Gln)	O'Driscoll et al. (2003); Heikkinen et al. (2005)
4	*BARD1*	Breast, Ovarian	DNA repair in oocytes; male infertility; small testes (mouse models)	Frameshift: c.2300_2301del (p.Val767fs)	Śniadecki et al. (2020); Ramus et al. (2015)
5	*CDH1*	Gastric, breast (lobular)	No direct infertility link	Missense, nonsense, frameshift (germline)	Garcia-Pelaez et al. (2023)
6	*CDK4*	Melanoma	No direct infertility link	Missense: c.70C>T (p.Arg24Cys)	Puntervoll et al. (2013)
7	*CDKN2A*	Melanoma, pancreatic	No direct infertility link	Missense, nonsense, deletion	Breen et al. (2025)
8	*CHEK1*	Breast, colon, prostate	Premature ovarian insufficiency; NOA	Missense: c.1169A>C (p.Tyr390Ser); c.1100delC (p.Thr367fs)	Guo et al. (2025); Kim et al. (2025); Emori and Boucher ZBolcun-Filas (2023); Ruotsalainen et al. (2024); Hashemi Karoii and Azizi HSkutella (2022)
9	*DICER1*	Pleuropulmonary blastoma, thyroid, ovarian (SCCOHT)	Male infertility; germ cell loss and meiotic arrest (mouse models)	Germline frameshift, nonsense, splice-site (e.g., c.2047_2050del, c.2555G>A); somatic RNase IIIb missense (p.E1705K, p.D1709N)	Schultz et al. (2024); Kommoss et al. (2023); Romero et al. (2011)
10	*DNMT3A*	Acute myeloid leukemia, myelodysplastic syndromes, lung cancer and breast cancer	Male infertility; germ cell loss	Somatic missense: p.R882H (AML); germline loss-of-function variants (clonal hematopoiesis)	Dura et al. (2022); Ye et al. (2023); Yang and Rau RGoodell (2015); Zhang et al. (2024b)
11	*EPCAM*	Colorectal (Lynch syndrome)	No direct infertility link	Frameshift: c.429_432del, c.491_494del, c.556_559dup	Lee et al. (2023); Boesch and Spizzo GSeeber (2018)
12	*ERCC1*	Cervical, lung, head and neck	Azoospermia	Missense: c.8092C>A (rs3212986)	Doll et al. (2013); Smith et al. (2014); Du et al. (2023); Ji et al. (2008)
13	*ESR1*	Breast, endometrial	Impaired spermatogenesis, endometriosis	c.454-397T>C (rs2234693, PvuII); c.454-351A>G (rs9340799, XbaI)	Dustin et al. (2019); Paskulin et al. (2013); Safarinejad and Shafiei NSafarinejad (2010)
14	*FANCA*	Leukemia, breast	Germ cell depletion; azoospermia; reduced testis weight; mosaic tubules; reduced ovary size; reduced follicles; no FANCD2 foci formation during meiosis	Biallelic: c.3348_3349del (p.Glu1116Aspfs19); c.3788_3790del (p.Ser1263del); c.887_890del (p.Glu296Valfs23); large deletions	Wong et al. (2003); Alavattam et al. (2016); Cheng et al. (2000); Dan et al. (2021); Abbasi and Rasouli (2017); Luo et al. (2023)
15	*FANCB*	Leukemia, breast	Male sterility; reduced testis weight; mosaic tubules; no FANCD2 foci formation during meiosis; unchanged MLH1 foci; subtly altered RAD51 dynamics	Biallelic loss-of-function; X-linked recessive	Alavattam et al. (2016); Kato et al. (2015); Fan et al. (2025); Du et al. (2015)
16	*FANCC*	Leukemia, head and neck, colon, lung, breast, renal and prostate cancer	Germ cell apoptosis; reduced testis weight; atrophic and mosaic tubules; reduced follicles; reduced ovary weight; no FANCD2 foci formation during meiosis	Biallelic: c.456 + 4A>T (splice-site); c.553C>T (p.Arg185Ter); c.1642C>T (p.Arg548Ter)	Nadler Jjbraun and Braun (2000); Whitney et al. (1996); Chen et al. (1996); Desai et al. (2026); Dörk et al. (2019)
17	*FANCD1 (Brca2)*	Breast, ovarian, prostate, pancreatic, gastric and thyroid cancer	Reduced ovary size. Reduced number of follicles, reduced germ cells and infertility	Frameshift: c.1796_1800del (p.Ser599Ter); c.3646_3649dup (p.Arg1217fs); c.3847_3848del (p.Val1283fs); c.5351dup (p.Asn1784fs); missense: c.7879A>T (p.Ile2627Phe)	Hartford et al. (2016); Sharan et al. (2004); Weinberg-Shukron et al. (2018); Xie et al. (2022)
18	*FANCD2*	Leukemia, breast and lung cancer	Reduced testis weight; mosaic tubules; reduced ovary size; reduced follicle number; no FANCD2 foci formation (as expected)	Biallelic loss-of-function; large deletions, frameshift	Alavattam et al. (2016); Houghtaling et al. (2003); Xie et al. (2024); Zhao et al. (2024a)
19	*FANCE*	Leukemia, liver, endometrial, head and neck cancer	Reduced testis weight; mosaic tubules; reduced ovary weight; reduced follicle number	Biallelic loss-of-function	Fu and Begum KOverbeek (2016); Fu et al. (2016); Zheng et al. (2023); Zhou et al. (2023)
20	*FANCF*	Ovarian	Smaller ovaries; reduced follicle numbers; reduced testis weight	Biallelic loss-of-function	Bakker et al. (2012)
21	*FANCG (Xrcc9)*	Adenocarcinoma and prostate	Reduced testis weight; mosaic tubules; reduced spermatozoa; reduced ovary size; reduced follicle number	Biallelic loss-of-function	Yang et al. (2001); Koomen et al. (2002); Gao et al. (2025); Gallmeier et al. (2006)
22	*FANCL (Pog/Phf9)*	Leukemia	Reduced testis weight; mosaic tubules; reduced spermatozoa; reduced ovary size; reduced follicle number; female sterility; allele- and age-dependent male infertility	Biallelic loss-of-function	Agoulnik et al. (2002); Lu and Bishop (2003a); Lu and Bishop (2003b); Frost et al. (2020)
23	*FANCM*	Breast, leukemia	Gonadal dysgenesis; azoospermia; NOA; POI; reduced testis weight; atrophic and mosaic tubules; reduced spermatozoa; reduced follicles; reduced ovary size	Biallelic nonsense: c.5101C>T (p.Gln1701Ter); frameshift: c.1491dup (p.Gln498fs); splice-site: c.4387–10A>G; C-terminal truncation causes cancer predisposition; C142R mutation does not	Kasak et al. (2018); Bakker et al. (2009); Yin et al. (2019); Figlioli et al. (2023); Pegoraro et al. (2026)
24	*FANCN (Palb2*)	Breast, pancreatic	Reduced testis weight; mosaic tubules; persistent DSBs and apoptosis in zygotene; decreased XY synapsis	Biallelic loss-of-function	Hartford et al. (2016); Simhadri et al. (2014); Tischkowitz and Xia (2010)
25	*FANCO (Rad51C)*	Ovarian, breast	Reduced testis weight; reduced spermatozoa; ovulation defect; fewer corpora lutea; fertile and infertile mice; spermatocytes: reduced MLH1, persistent γ-H2AX, reduced RAD51; arrest in meiosis I; chromosome fragmentation; oocytes: precocious separation of sister chromatids at metaphase II	Biallelic loss-of-function; knockout/neo-hypomorph analyzed; embryonic lethal in complete knockout	Kuznetsov et al. (2007); Somyajit and Subramanya (2012); Rein et al. (2025)
26	*FANCP (Slx4/Btbd12)*	Leukemia (Fanconi anemia) and breast cancer	Meiotic defects; POI; reduced testis weight; reduced spermatozoa; no follicles; females sterile; males near-sterile; increased MLH1/3 foci; unchanged number of chiasmata	Biallelic loss-of-function	Holloway et al. (2011); Crossan et al. (2011); Bakker et al. (2013); Spinella et al. (2015)
27	*FANCS* (*Brca1*)	Breast and ovarian cancer	Reduced testis weight; no spermatozoa; sterile males; fertile females; spermatocytes: reduced RAD51 foci; normal DMC1 foci; normal synapsis; lack of chiasmata and MLH1 in males; pachytene arrest; oocytes: MLH1 normal	Biallelic loss-of-function; Brca1^−/−^ p53+/−mouse model	Xu et al. (2003); Petrucelli et al. (1993)
28	*FANCU (Xrcc2)*	Breast and lung cancer	Reduced testis weight; no spermatozoa; reduced follicles; male sterility	Biallelic loss-of-function	Yang et al. (2018); Gong et al. (2024); Park et al. (2012)
29	*FANCV* (*Rev7/Mad2l2*)	Leukemia, lung, ovarian and skin cancer	Reduced testis weight; atrophic tubules; no spermatozoa; PGCs lost during migration; atrophic ovaries; no follicles; sterile	Biallelic loss-of-function	Watanabe et al. (2013); Maggs and McVey (2025)
30	*FANCW (Rfwd3)*	Ovarian, Fanconi anemia	Meiosis; POI; few spermatozoa; atrophic ovaries; no follicles; male and female sterility	Biallelic loss-of-function	Knies et al. (2017); Taylor et al. (2024)
31	*FAAP20*	Leukemia, breast	Reduced testis weight; smaller ovaries; reduced follicle number	Biallelic loss-of-function	Zhang et al. (2015)
32	*USP1*	Ovarian and pancreatic cancer	Reduced testis weight; atrophic tubules; reduced oocyte number; male sterility	Biallelic loss-of-function	Kim et al. (2009); Li et al. (2025); Sonego et al. (2019)
33	*UAF1*	Lung cancer	Reduced testis weight; reduced oocyte number	Biallelic loss-of-function; Uaf1+/−mouse model	Park et al. (2013); Chen et al. (2011)
34	*FGFR3*	Bladder, multiple myeloma	Mutant sperm production	Activating missense: c.746C>G (p.Ser249Cys) in bladder cancer; c.1138G>A (p.Gly380Arg) in achondroplasia	Ascione et al. (2023); St-Germain et al. (2009); Striedner et al. (2024)
35	*FH*	Kidney (HLRCC), uterine leiomyomas	Affects implantation, endometrial function	Missense: c.1431_1433dupAAA; c.698G>A (p.Arg233His); frameshift	D'Indinosante et al. (2025); Schwartz et al. (2023)
36	*FLCN*	Renal (Birt-Hogg-Dubé)	No direct infertility link	Frameshift: c.1285dupC; c.1533_1536delGAGA	Schmidt LSLinehan and Linehan (2018)
37	*GREM1*	Colorectal (polyposis)	No direct infertility link	40 kb duplication upstream of GREM1	Zhu et al. (2023)
38	*H3F3A*	Pediatric glioblastoma, giant cell tumors and lung cancer	Male infertility (mouse models)	Somatic missense: c.83A>T (p.Lys28Met); c.103G>A (p.Gly35Arg)	Park et al. (2016); Presneau et al. (2015); Bush et al. (2023)
39	*H3F3B*	Pediatric glioblastoma and colorectal cancer	Testicular atrophy and germ cell loss	Somatic missense: c.83A>T (p.Lys28Met)	Yuen et al. (2014); Gunes SEsteves and Esteves (2021); Ayoubi and Mahjoubi FMirzaei (2017)
40	*HOXB13*	Prostate and ovarian cancer	Ejaculatory dysfunction; prostate gland malformation	Missense: c.251G>A (p.Gly84Glu); c.650G>T (p.Arg217Leu)	Ewing et al. (2012); Miao et al. (2007)
41	*KASH5*	Emerging cancer link	Meiotic telomere attachment, azoospermia and POI	L535Q, c.310C>T (p.Arg104Ter); c.1071_1072del (p.Pro358Serfs*2)	Bentebbal et al. (2021); Hou et al. (2023); Zhang et al. (2022)
42	*KIT*	Colorectal cancer	Oligospermia	Activating missense: c.2446A>T (p.Asp816Tyr); c.2447T>A (p.Leu816Tyr)	Küçükköse et al. (2022); Shi et al. (2016); Galan et al. (2006)
43	*LGR4*	Emerging cancer link	Gonadogenesis; POI	Biallelic loss-of-function: c.776T>C (p.Leu259Pro); c.1699C>T (p.Arg567Ter)	Ordaz-Ramos et al. (2021); Pan et al. (2014)
44	*LZTR1*	Schwannomatosis, Noonan syndrome	Gonadal dysfunction, azoospermia, oligozoospermia	Missense: c.848G>A (p.Arg283Gln)	Motta et al. (2019); Orsolini et al. (2024)
45	*MAX*	Neuroendocrine cancer	No direct infertility link	Missense, nonsense, frameshift	Freie et al. (2025)
46	*MCMDC2*	RecQ-related cancer risk	Meiotic arrest; POI	Biallelic loss-of-function: c.1222C>T (p.Arg408Ter); c.1783C>T (p.Arg595Ter)	Cohen et al. (2026)
47	*MEN1*	Endocrine tumors	Pituitary tumors may affect gonadal axis	Frameshift: c.249_252del; nonsense: c.1546C>T (p.Arg516Ter)	Schuppe et al. (1999)
48	*MET*	Breast and Ovarian cancer	No direct infertility link	Missense: c.3029T>C (p.Met1010Thr)	Tovar and Graveel (2017)
49	*MITF*	Cutaneous melanoma	No direct infertility link	Missense: E318K	Guhan et al. (2020)
50	*MLH1*	Colorectal, endometrial (Lynch)	Aneuploid oocytes, spontaneous pregnancy loss; NOA; oligozoospermia; azoospermia	Missense: c.74T>C (p.Ile25Thr); promoter methylation; c.2152G>A (p.Ala718Thr)	Dowty et al. (2013); Singh et al. (2021)
51	*MLH3*	Colorectal	Azoospermia; oligozoospermia	Missense: c.2152G>A (p.Ala718Thr); c.2531C>T (p.Pro844Leu)	Singh et al. (2021); Korhonen et al. (2008); Markandona et al. (2015); Nawaz et al. (2021)
52	*MRE11A*	Breast and ovarian cancer	Spermatogenic failure	Biallelic: c.14C>T (p.Ala5Val); c.817G>A (p.Gly273Arg), p.E506*	Hu et al. (2020); Elkholi et al. (2021)
53	*MSH2*	Colorectal, endometrial (Lynch)	Infertility possible in Lynch patients	c.2039G>A (p.Arg680Gln); c.2048G>T (p.Gly683Val); c.354T>A (p. Y118*)	Zhao et al. (2026); Zhong et al. (2024)
54	*MSH5*	Various	Meiotic arrest; oligozoospermia; POI	c.85C>T (p.Pro29Ser); c.2392C>T (p.Arg798Cys) c.1126del	Gong et al. (2022); Guo et al. (2017); Liu et al. (2017)
55	*MSH6*	Colorectal, endometrial (Lynch)	DNA repair in gametes; no major fertility defect	Frameshift: c.2569_2572del (p.Asp857fs)	Werf – ’t et al. (2025); Perry et al. (2014)
56	*MUTYH*	Colorectal (MAP)	No direct infertility link	Biallelic: c.494A>G (p.Tyr165Cys); c.1145G>A (p.Gly382Asp)	Georgeson et al. (2022)
57	*NBN*	Breast, lymphoma	Meiotic synapsis failure	Founder: c.657_661del5 (p.Lys219fs); biallelic cause Nijmegen breakage syndrome, (c.442A>G, p. (Thr148Ala)	Fiévet et al. (2020); Belhadj et al. (2023)
58	*NF1*	Nerve sheath tumors and others	Pubertal delay; subfertility; cryptorchidism	c.5624 C > A p. (Ser1875*)	Philpott et al. (2017); Pacot et al. (2024)
59	*NF2*	Schwannoma, meningioma	No direct infertility link	Nonsense and frameshift mutations	Xu and Yin SShu (2024)
60	*PHOX2B*	Neuroblastoma	No direct infertility link	Missense: c.422G > A (p.Arg141Gln)	Wu et al. (2024)
61	*PMS2*	Colorectal, endometrial (Lynch)	abnormal chromosome synapsis in meiosis in mice	Pms2-deficinet mice	Baker et al. (1995); Andini et al. (2023)
62	*PRDM1*	Gastric cancer	Gonadogenesis; Pregnancy loss	PR-domain deletion	Qin et al. (2025); Du et al. (2020)
63	*PTCH1*	Breast cancer	hypogonadism in both males and females	Ptch1 deletion; c.3907C > T c.3538C > T	Ren et al. (2019); Wang et al. (2019)
64	*PTEN*	Various cancers	Ovarian function regulation; follicular quiescence; POI; sperm maturation	Missense, nonsense, frameshift (Cowden syndrome)	Cummings et al. (2023); Xu and Washington AMHinton (2014); Xue et al. (2023)
65	*RAD51*	Breast, ovarian	Reduced testis weight; RAD51 labels meiotic DSBs; siRNA injection against RAD51 leads to p53-dependent apoptosis and reduced MLH1 foci	Missense, frameshift (Fancr); embryonic lethal in complete knockout	Qin et al. (2022); Wang et al. (2022)
66	*RB1*	Retinoblastoma	Testicular atrophy and infertility	Conditional deletions	Iacovacci et al. (2025); Nalam et al. (2008)
67	*RECQL4*	Rothmund-Thomson syndrome	Chromosomal instability and infertility; reproductive aging	Biallelic variant	Kohzaki et al. (2021); Martin-Giacalone et al. (2022)
68	*RET*	Various cancers	No direct infertility link	S891L; M918T	Zhou et al. (2022)
69	*SMAD4*	Colorectal and pancreatic cancer	No effect on fertility	Deletion in granulosa cells of *Smad4;* 18q21 loss in *SMAD4*	Woodford-Richens et al. (2001); Yao et al. (2024)
70	*SMARCA4*	Breast and lung cancer	Essential in meiosis	Enhanced phosphorylation levels of S613, S695, S699, and S1417	Peng et al. (2021); Kim and Fedoriw AMMagnuson (2012)
71	*SPRED1*	Legius syndrome	No direct infertility link	Frameshift (p.Ile60Tyrfs*18) and missense (p.Pro422Arg)	Bianchi et al. (2015)
72	*STK11*	Various cancer	Subfertility due to Peutz-Jeghers Syndrome (PJS)	splice site 375-1delC	Rutherford et al. (2024); Huang et al. (2025)
73	*STRA8*	Emerging cancer link	Meiosis; POI	Biallelic loss-of-function: c.198_199del (p.Val67Valfs*20); c.688C>T (p.Arg230Ter)	Ma et al. (2018); França MMMendonca and Mendonca (2022)
74	*TP53*	Li-Fraumeni (multiple cancers)	Germ cell apoptosis; gonadal failure risk; POI	Missense: c.542G>A (p.Arg181His); c.524G>A (p.Arg175His); c.818G>A (p.Arg273His)	Chen et al. (2022a); Paskulin et al. (2012)
75	*TP63*	EEC syndrome, breast	Müllerian duct anomalies; POI	Missense: c.1010G>A (p.Arg337His); c.*374G>A	Luan et al. (2021); Wang et al. (2016)
76	*TSHR*	Thyroid cancer	Possible infertility	Activating: c.1609G>A (p.Gly537Arg); rs2268458 intronic polymorphism	Xu et al. (2025); Naghibi and Miresmaeili (2022)
77	*UAF1*	Leukemia, prostate	Reduced testis weight; spermatogenic defect	Biallelic loss-of-function; Uaf1+/−mouse model	Wang et al. (2024b)
78	*USP1*	Various cancer	Reduced testis weight; atrophic tubules; reduced oocyte number; male sterility	Biallelic loss-of-function	Zhao et al. (2020); Mazloumi and Walden (2025); Kim (2026)
79	*VHL*	Renal, hemangioblastoma	Male infertility related to VHL disease; small testes, obstructive azoospermia	Missense: c.194C>G (p.Ser65Ter); c.464-1G>A; p.); c.481C>T (p.Arg161Ter); large deletions	Scafa et al. (2023); Kim WYKaelin and Kaelin (2004)
80	*WT1*	Wilms tumor; various cancer	Gonadal dysgenesis; NOA; cryptorchidism; premature ovarian failure	1238G > T; R413M; rs373176048	Nian et al. (2025); Nathan et al. (2017)
81	*XRCC1*	Various cancer	Azoospermia; abnormal spermatogenesis	Arg399Gln polymorphism	Lv et al. (2020); Wang et al. (2024c)

Data based on The Human Protein Atlas (https://www.proteinatlas.org/), Alliance of Genome Resources (https://www.alliancegenome.org/), and published literature.

#### 3.2.1 Preclinical evidence from mouse models

Mouse models reveal that homozygous loss of key DNA repair genes often causes complete sterility, providing mechanistic validation for human infertility phenotypes. As summarized in Table 2, *Brca2* deficiency leads to meiotic impairment and infertility (Sharan et al., 2004), while *Brca1* knockout leads to testicular atrophy and meiotic defects (Simhadri et al., 2014). *Atm*-deficient mice exhibit small testes and infertility (Barlow et al., 1998), and *Fancm* knockout results in small testes with reduced germ cells (Tsui et al., 2023). Among mismatch repair genes, *Mlh1* loss causes spermatogenic arrest (Edelmann et al., 1996), whereas *Msh6* deficiency shows no fertility defect (Mukherjee and Ridgeway Adlamb, 2010), suggesting compensatory mechanisms. *Trp53* knockout mice demonstrate abnormal spermatogenesis and increased germ cell apoptosis (Yao et al., 2024), while *Chek2* deficiency exhibits no reproductive phenotype (Li et al., 2024). Developmental regulators *Wt1* and *Hoxb13* induce structural defects matching their expression patterns (Wang et al., 2013; Post Lcinnis and Innis, 1999).

**TABLE 2:** Preclinical evidence from mouse models.

Gene category	Gene name	Protein function	Mouse model phenotype	References
DNA Repair	*Atm*	DSB Repair	Small testes, infertile (Hom)	Barlow et al. (1998); Tal et al. (2018)
​	*Brca1*	HR Repair	Testicular atrophy (Hom)	Xu et al. (2003); Liu Ylu and Lu (2020)
​	*Brca2*	Meiotic recombination	Complete sterility (Hom)	Sharan et al. (2004)
​	*Fancm*	Crosslink repair	Small testes (Hom)	Bakker et al. (2009); Tsui et al. (2023)
Mismatch Repair	*Mlh1*	Meiotic crossover	Spermatogenic arrest (Hom)	Edelmann et al. (1996); Shrestha et al. (2021)
​	*Msh6*	Mismatch repair	No defect reported	Shrestha et al. (2021)
Tumor Suppressor	*Trp53 (p53)*	Genome stability	Abnormal spermatogenesis (Hom)	Eischen (2016); Zalzali et al. (2018)
​	*Chek2*	Cell cycle checkpoint	No phenotypes reported	Bahassi el et al. (2009)
Developmental Regulators	*Wt1*	Gonadal development	Testis hypoplasia (Hom/het)	Wang et al. (2013); Chen et al. (2022b)
​	*Hoxb13*	Prostate morphogenesis	Gland malformation (Hom)	Post Lcinnis and Innis (1999); Economides and Capecchi (2003)

Reported mouse models with reproductive male phenotype and predisposition to cancer were based on the Mouse Genome Informatics database (https://www.informatics.jax.org/).

#### 3.2.2 Clinical evidence in humans

Female Infertility (POI and DOR): The critical role of *BRCA2* in ovarian development has been elucidated by Weinberg-Shukron et al. (Weinberg-Shukron et al., 2018), who demonstrated that biallelic hypomorphic *BRCA2* variants cause isolated XX ovarian dysgenesis manifesting as absence of spontaneous pubertal development and primary amenorrhea. Cells from affected sisters expressed only 14% of normal BRCA2 protein levels and showed reduced recruitment of RAD51 to double-stranded DNA breaks. Similarly, Caburet et al. (Caburet et al., 2020) reported a homozygous missense c.8524C>T/p.R2842C-*BRCA2* variant in a patient with isolated POI without cancer or Fanconi anemia traits, with functional studies showing intermediate levels of chromosomal breaks and partial HR complementation. Regarding *BRCA1*, biallelic frameshift mutations (c.470_471del; c.791_794del) were recently identified in a patient with isolated diminished ovarian reserve without Fanconi anemia, demonstrating residual DNA repair activity via a truncated del11q isoform (Helbling-Leclerc et al., 2024).

Beyond individual gene reports, a prospective study of 120 patients with unexplained diminished ovarian reserve (DOR) using a large custom targeted next-generation sequencing panel identified a genetic etiology in 24.2% of cases. Genes belonged to distinct pathways: metabolism and mitochondria (29.7%), follicular growth (24.3%), DNA repair and meiosis (18.9%), aging (16.2%), ovarian development (8.1%), and autophagy (2.7%). Five genes were recurrently mutated: LMNA, ERCC6, SOX8, POLG, and BMPR1B. Additionally, six genes previously identified in single families with POI (GNAS, TGFBR3, XPNPEP2, EXO1, BNC1, ATG) were found in DOR patients, highlighting their role in maintaining ovarian reserve. Notably, no pregnancy was achieved when meiosis/DNA repair genes (including EXO1) were involved, suggesting severely impaired oocyte quality in this subgroup (Lafraoui et al., 2024).

Male Infertility: For *BRCA2*, the common variant N372H has been associated with idiopathic male infertility with azoospermia or severe oligozoospermia (Zhoucun et al., 2006). *ATM* heterozygotes show non-obstructive azoospermia with defects tightly linked to meiotic gene expression (Li et al., 2013). Biallelic FANCM mutation was first identified in a Finnish family with POI (Fouquet et al., 2017) and later confirmed in a large cohort of patients with POI (Heddar et al., 2022). Subsequently, biallelic FANCM mutations were identified in patients with non-obstructive azoospermia (NOA) (Kasak et al., 2018; Yin et al., 2019). Table 3 summarizes the human fertility impacts, associated cancer predispositions, and clinical actionability for the major genes discussed in this section.

**TABLE 3:** Human clinical correlations and actionability.

Gene category	Gene name	Human fertility impact	Cancer predisposition	Clinical actionability
DNA Repair	*ATM*	Oligospermia	Lymphoma, breast	Sperm banking recommended
​	*BRCA1*	Azoospermia rare	Breast, ovarian, prostate	Micro-TESE may be considered
​	*BRCA2*	89% sperm retrieval success	Pancreatic, breast	Prioritize early fertility preservation
​	*FANCM*	Not well characterized	Leukemia	Consider testicular biopsy
Mismatch Repair	*MLH1*	Possible subfertility	Lynch syndrome cancers	Screen for cryptorchidism
​	*MSH6*	Unaffected	Endometrial, colorectal	Routine fertility evaluation
Tumor Suppressor	*TP53*	Germ cell apoptosis	Li-Fraumeni spectrum	Cryopreservation pre-chemo
​	*CHEK2*	Controversial data	Breast, colon	Baseline semen analysis
Developmental Regulators	*WT1*	Severe infertility	Wilms tumor	Early andrology consult
​	*HOXB13*	Ejaculatory dysfunction	Prostate cancer	Evaluate semen parameters

Expression data based on The Human Protein Atlas (https://www.proteinatlas.org/) with the highest levels of expression listed first.

#### 3.2.3 Integrated mechanistic synthesis

The intricate relationship between DNA repair genes, fertility, and cancer susceptibility is exemplified by several critical genes whose dysfunction disrupts both germline genomic stability and somatic tumor suppression (Panier and Wang, 2024). Among these, *BRCA1, BRCA2, ATM, and CHEK2* stand out for their pleiotropic roles in maintaining meiotic fidelity and preventing malignant transformation (Liu et al., 2025).

*BRCA1*, a cornerstone of homologous recombination (HR) repair, is indispensable for resolving DNA double-strand breaks (DSBs) during meiosis (Olmos et al., 2025). In its absence, oocytes and spermatocytes accumulate unrepaired DSBs, leading to premature ovarian insufficiency (Oktay et al., 2010) and *BRCA1/2* are required for the completion of embryogenesis (Tulay et al., 2017). These reproductive deficits mirror *BRCA1’s* role in carcinogenesis, where HR deficiency drives genomic instability in breast, ovarian, and prostate tissues. The shared mechanism lies in failed DSB repair: during meiosis, unresolved breaks trigger germ cell apoptosis, while in somatic cells, they promote oncogenic rearrangements (Krais and Johnson, 2020).

*BRCA2*, another HR pathway effector, exhibits an even more pronounced dual phenotype. The protein stabilizes RAD51 filaments during meiotic recombination, and its loss disrupts crossover formation in gametes while permitting mitotic errors in somatic cells (Deveryshetty et al., 2025). This reproductive failure parallels *BRCA2*-associated pancreatic cancer and melanoma, where somatic HR defects foster aggressive tumorigenesis (Mekonnen and Yang HShin, 2022).

**FIGURE 1:**
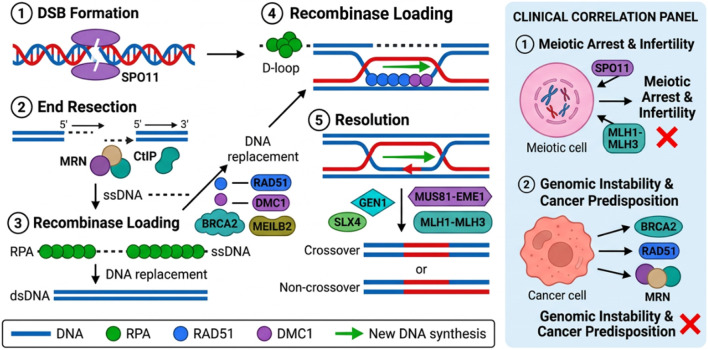
Molecular steps of homologous recombination repair. This figure illustrates the sequential molecular events of homologous recombination (HR) repair of DNA double-strand breaks (DSBs), a pathway essential for both meiotic fidelity in germ cells and genomic stability in somatic tissues. Step 1 (DSB Formation): SPO11 topoisomerase-like protein introduces programmed DSBs during meiotic prophase I. Step 2 (End Resection): The MRE11-RAD50-NBS1 (MRN) complex with CtIP processes DNA ends to generate 3′ single-stranded DNA overhangs, which are initially coated by replication protein A (RPA). Step 3 (Recombinase Loading): BRCA2 and MEILB2 mediate the replacement of RPA with RAD51 and DMC1 recombinases, forming nucleoprotein filaments. BRCA2 stabilizes RAD51 filaments; its dysfunction causes meiotic arrest and azoospermia. Step 4 (Strand Invasion): The RAD51/DMC1-coated filament searches for homologous DNA sequences and invades double-stranded DNA to form a displacement loop (D-loop). Step 5 (D-loop Formation and DNA Synthesis): DNA polymerase extends the invading strand using the homologous template. Step 6 (Resolution): Holliday junctions are resolved by resolvases (GEN1, SLX4, MUS81-EME1) with MLH1-MLH3 marking crossover sites. Clinical Consequences Panel: Failure at any step leads to meiotic arrest with germ cell apoptosis (infertility) and genomic instability (cancer predisposition).

*ATM*, a master regulator of DSB signaling, illustrates how cell cycle checkpoint defects impair both reproduction and immunity (Weitering et al., 2021) (Figure 1). Males with *ATM* mutations exhibit spermatogenic arrest at the pachytene stage, as unrepaired meiotic DSBs activate apoptotic pathways (Pacheco et al., 2015). Concurrently, *ATM* deficiency predisposes to leukemia and lymphoma due to unchecked proliferation of DNA-damaged lymphocytes (Lee, 2025). The common thread is *ATM*’s role in phosphorylating *p53 and CHK2*, which coordinate meiotic arrest and somatic cell apoptosis (Stracker et al., 2013).

*CHEK2*, a downstream target of *ATM*, underscores the interplay between DNA damage responses and hormonal regulation (Smith et al., 2010). Mutations in *CHEK2* cause premature ovarian insufficiency by inducing p53-mediated oocyte depletion, while concurrently increasing risks of breast and colon cancer via defective cell cycle arrest (Emori and Boucher Zbolcun-Filas, 2023; Mundt et al., 2023). This dual pathology arises because *CHEK2* phosphorylates both meiotic cohesins (required for chromosome segregation) and somatic tumor suppressors like *BRCA1* (Stolz et al., 2010).

The integration of preclinical models and clinical data underscores a fundamental principle: genes critical for maintaining genomic stability exert pleiotropic effects across germline and somatic tissues. The stark infertility observed in mouse knockouts provides mechanistic validation for the subfertility phenotypes seen in human carriers, while the nuanced differences-such as the dissociation between cancer risk and fertility in some MMR genes-highlight the complexity of translating molecular pathways into clinical outcomes. This evidence solidifies the biological link between gametogenesis failure and cancer susceptibility, establishing a compelling rationale for the genetic screening and multidisciplinary oncofertility management strategies discussed in subsequent sections.

### 3.3 Cell cycle regulators

The tumor suppressor genes *TP53* and *PTEN* play pivotal roles in maintaining genomic integrity and regulating cell proliferation, with germline mutations in these genes leading to cancer predisposition syndromes-Li-Fraumeni syndrome (LFS) and Cowden syndrome, respectively (Rocca et al., 2022). Beyond their well-documented oncogenic effects, emerging evidence highlights their critical involvement in reproductive dysfunction, linking defective cell cycle regulation to gonadal failure and endometrial hyperplasia, ultimately contributing to infertility (Kamal et al., 2016).

**FIGURE 2:**
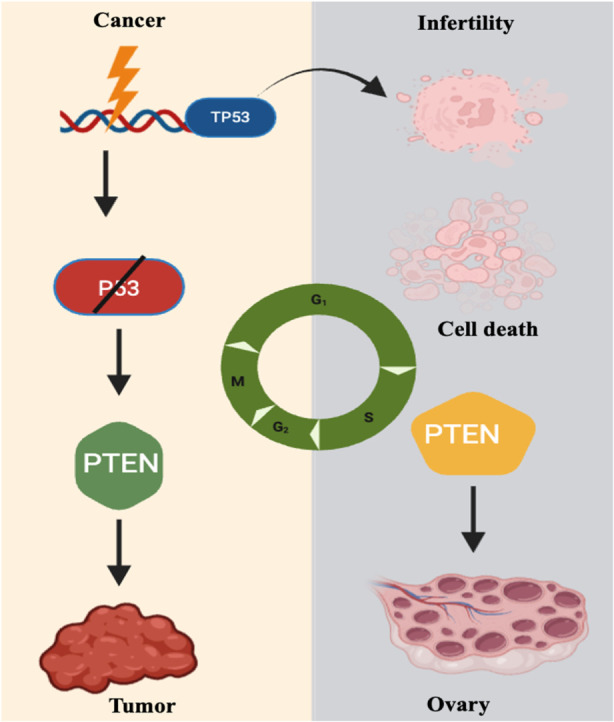
*TP53* and *PTEN* at the cancer-infertility crossroads. The figure shows overlapping regulators (TP53, PTEN) and cell cycle phases (G1/S/G2/M) connecting cancer (especially ovarian) with infertility, suggesting common pathways in cell death and dysfunction. This illustrates how dysregulated cell cycle checkpoints can simultaneously permit somatic tumorigenesis and trigger germ cell apoptosis, directly linking carcinogenesis to gonadal failure.

*TP53*, encoding the p53 protein, serves as a master regulator of stress responses, orchestrating cell cycle arrest, DNA repair, or apoptosis in damaged cells (Wang H. et al., 2023). In LFS, germline *TP53* mutations result in loss of controlled p53 activity, leading to genomic instability and increased susceptibility to early-onset malignancies, including sarcomas, breast cancer, and brain tumors (Rocca et al., 2022) (Figure 2). However, the impact of *TP53* dysfunction extends beyond somatic tissues, significantly affecting germ cell viability (Timmerman et al., 2021). Studies demonstrate that mutant p53 triggers excessive apoptosis in oocytes and spermatogonia, depleting ovarian reserves and impairing spermatogenesis (Li et al., 2024). Male carriers for LFS often present with oligospermia due to germ cell attrition (Matwiejczyk, 2020). Mechanistically, p53’s role in eliminating damaged germ cells becomes dysregulated in LFS, where unchecked apoptotic signaling leads to premature gonadal failure (Li et al., 2024). Similarly, *PTEN*, a key negative regulator of the PI3K/AKT/mTOR pathway, is frequently mutated in Cowden syndrome, predisposing individuals to endometrial, thyroid, and breast cancers (Dragoo et al., 2021). PTEN’s tumor-suppressive function hinges on its ability to restrain PI3K-mediated proliferative signaling, and its loss leads to hyperactivation of AKT, driving uncontrolled cell growth (Wang et al., 2025). In the context of reproduction, *PTEN* deficiency disrupts endometrial homeostasis, resulting in hyperplasia and impaired implantation, a common cause of infertility in affected women (Eritja et al., 2021). Additionally, PTEN plays a crucial role in follicular quiescence, where its loss accelerates primordial follicle activation, prematurely depleting ovarian reserves and contributing to premature ovarian aging (Maidarti and Anderson RATelfer, 2020). Mouse models with *Pten* deletions exhibit rapid follicle exhaustion, mirroring the diminished ovarian reserve observed in Cowden syndrome patients (Hollander and Blumenthal GMDennis, 2011).

### 3.4 Epigenetic modifiers

Germline mutations in epigenetic regulators DNMT3A and MTHFR create a fascinating biological paradox, where defects in DNA methylation and folate metabolism pathways simultaneously predispose to both cancer and reproductive dysfunction (Fardous AMHeydari and Heydari, 2023). The DNA methyltransferase DNMT3A illustrates this duality through its tissue-specific effects on epigenetic regulation (Tóth et al., 2025). In hematopoietic stem cells, loss-of-function mutations drive leukemogenesis by causing genome-wide hypomethylation that destabilizes transcriptional programs and promotes malignant transformation, particularly in acute myeloid leukemia and myelodysplastic syndromes (Benetatos and Vartholomatos, 2018). This oncogenic effect stems from DNMT3A’s crucial role in maintaining proper methylation patterns during cell differentiation (Challen et al., 2012). Remarkably, the same gene exhibits opposite epigenetic consequences in male germ cells, where mutations lead to localized hypermethylation at critical imprinted loci like H19 and MEST during spermatogenesis (Kerjean et al., 2000). This aberrant methylation patterning disrupts the precise epigenetic reprogramming required for proper sperm development, resulting in oligospermia and reduced fertility (McSwiggin and O'Doherty, 2018). The contrast between somatic hypomethylation and germline hypermethylation highlights the context-dependent nature of DNMT3A function, where the same molecular defect produces divergent clinical outcomes through distinct mechanistic pathways (Poeta et al., 2020). Similarly, MTHFR polymorphisms impair folate metabolism in ways that simultaneously increase cancer risk while compromising reproductive success (Ledowsky and Steel ASchloss, 2021).The enzyme’s role in regulating methyl group availability creates a metabolic bottleneck when compromised, leading to genome-wide hypomethylation in rapidly dividing somatic cells that promotes malignant transformation, particularly in colorectal epithelium (Zhao N. et al., 2024). This same metabolic disruption proves catastrophic for early embryonic development, where proper methylation patterning is essential for normal gene expression and chromosomal stability (Breton-Larrivée and Elder EMcGraw, 2019).

## 4 Convergent pathways: DNA repair, epigenetics, and hormonal crosstalk

Three interconnected pathways-DNA repair (MLH1), epigenetic regulation (DNMT3A), and hormonal signaling (ESR1)-explain most shared infertility-cancer phenotypes. Their convergence underscores the need for multidisciplinary interventions targeting both reproductive and oncologic outcomes. The maintenance of genomic stability is paramount for both successful reproduction and prevention of malignant transformation (Yao and Dai, 2014). Germline mutations in genes critical for meiotic fidelity (MLH1, MSH6) and telomere homeostasis (DKC1) exemplify how defects in fundamental cellular processes can simultaneously impair fertility and promote carcinogenesis (Rosta, 2022). These dual outcomes arise from shared molecular pathways that, when compromised, manifest differently in germ cells versus somatic tissues.

### 4.1 DNA mismatch repair genes (*MLH1, MSH6*): meiotic defects and microsatellite instability

**FIGURE 3:**
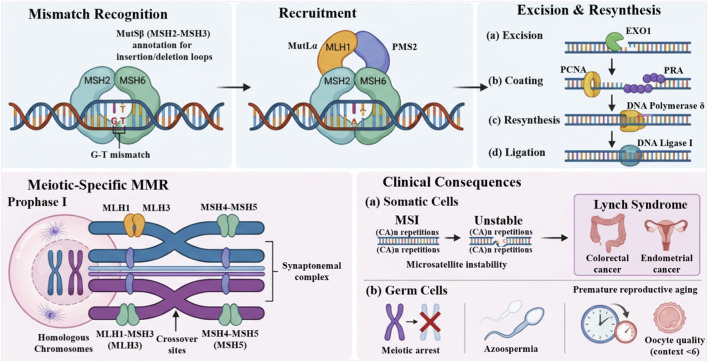
Schematic representation of mismatch repair. This figure depicts the molecular mechanism of DNA mismatch repair (MMR), demonstrating how this pathway maintains genomic integrity in somatic cells while ensuring proper meiotic chromosome segregation in germ cells. Step 1 (Mismatch Recognition): MutSα complex (MSH2-MSH6) recognizes single-base mismatches and small insertion-deletion loops. MutSβ (MSH2-MSH3) recognizes larger loops. MSH6 defects spare fertility, while MSH2 mutations cause more severe reproductive phenotypes. Step 2 (MutL Recruitment): MutLα complex (MLH1-PMS2) binds to MutSα. During meiosis, MLH1 also forms MutLγ (MLH1-MLH3) essential for crossover formation. MLH1 loss causes complete spermatogenic failure and azoospermia; MLH3 mutations cause oligozoospermia. Step 3 (Excision): EXO1 (exonuclease 1) is recruited to excise the mismatch-containing DNA strand, facilitated by PCNA and RPA. Step 4 (Resynthesis and Ligation): DNA polymerase δ resynthesizes the excised region using the intact complementary strand as template; DNA ligase I seals the nick. Meiotic Crossover: During prophase I of meiosis, MLH1-MLH3 foci mark crossover sites (chiasmata), ensuring proper chromosome segregation. Loss of MLH1 or MSH5 disrupts this process, causing aneuploid gametes. Clinical Consequences: Somatic MMR defects cause microsatellite instability (MSI) and Lynch syndrome cancers (colorectal, endometrial, ovarian). Germline MMR defects cause meiotic arrest, azoospermia, oligozoospermia, and premature reproductive aging. Key genes labeled: MSH2/MSH6 (recognition), MLH1/PMS2 (MutL complex), EXO1 (excision), MLH1/MSH5 (meiotic crossover).

The DNA mismatch repair (MMR) system, orchestrated by MLH1 and MSH6, plays a pivotal role in maintaining genomic integrity during both meiosis and mitosis (Figure 3) (Pećina-Šlaus et al., 2020). In somatic cells, MMR proteins correct replication errors, particularly in repetitive microsatellite regions (Aquilina and Bignami, 2001). Germline mutations in these genes cause Lynch syndrome, characterized by microsatellite instability (MSI) and markedly elevated risks of colorectal, endometrial, and ovarian cancers (Mitric et al., 2023). (Dörk et al., 2019)

Remarkably, these same MMR proteins are indispensable for proper meiotic chromosome segregation. During prophase I, *MLH1* marks sites of crossover formation, ensuring proper chiasma frequency and distribution (Falque et al., 2007). Loss of *MLH1* or *MSH6* function disrupts this process, leading to aberrant meiotic recombination and subsequent production of aneuploid gametes (Singh et al., 2021). Mouse models recapitulate this phenomenon, with *Mlh1* knockout females exhibiting complete meiotic arrest and males showing oligospermia.

### 4.2 *DKC1* and telomere biology: linking ovarian aging to epithelial cancers

**FIGURE 4:**
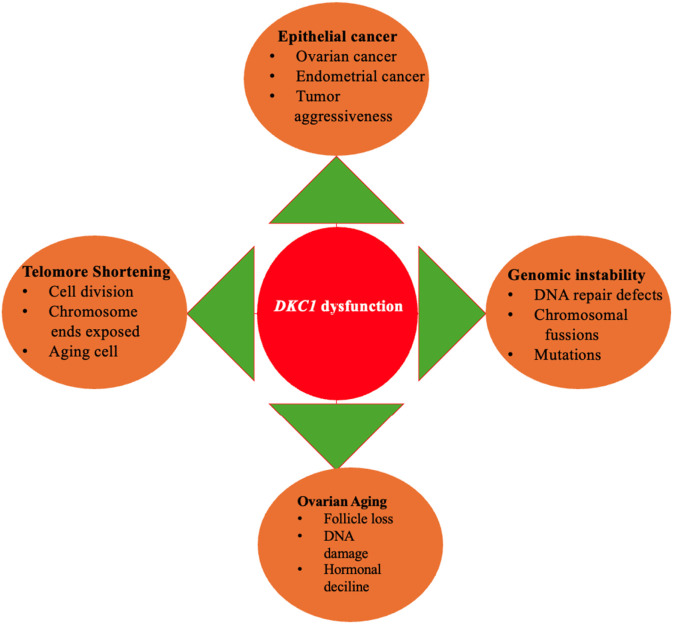
Interlinked factors in ovarian aging and epithelial cancer risk. This figure illustrates the connections between telomere shortening, *DKC1* dysfunction, and ovarian aging, highlighting how genomic instability and DNA damage contribute to both accelerated ovarian decline and increased risk of aggressive epithelial cancers (e.g., ovarian, endometrial). The overlap underscores shared molecular pathways in aging and carcinogenesis. The overlap underscores how fundamental processes of cellular aging, like telomere attrition, can simultaneously drive reproductive decline and carcinogenesis in rapidly dividing tissues.

The DKC1 gene encodes dyskerin, a pseudouridine synthase essential for telomerase RNA stabilization and telomere maintenance (Garus AAutexier and Autexier, 2021). Germline DKC1 mutations underlie dyskeratosis congenita (DC), a disorder characterized by progressive telomere shortening that drives both hematopoietic failure and epithelial carcinogenesis (Savage, 2022). Parallel telomere dysfunction in the germline creates equally severe reproductive consequences (Figure 4). Oocytes, which remain arrested in meiotic prophase I for decades, are exquisitely sensitive to DNA damage accumulation (Ferreira et al., 2023). DKC1 mutations accelerate telomere erosion in ovarian follicles, precipitating premature oocyte depletion and early menopause (Zhu and Xu WLiu, 2023). Male carriers similarly exhibit spermatogenic failure due to apoptosis of telomere-deficient spermatogonia. This shared vulnerability of germ cells and epithelial progenitors to telomere dysfunction illustrates how fundamental processes of cellular aging can simultaneously drive both reproductive decline and carcinogenesis (Pohl et al., 2021).

### 4.3 Hormonal dysregulation

Estrogen receptor alpha gene (ESR1) mutations disrupt estrogen signaling, causing progesterone resistance and impaired endometrial receptivity, leading to implantation failure and infertility (Yilmaz BDBulun and Bulun, 2019). In endometriosis, hyperactive ERα promotes inflammatory lesions that damage ovarian tissue and fallopian tubes, while in breast tissue, the same mutations drive cancer via unregulated proliferation (Guo, 2020). This dual effect stems from tissue-specific dysregulation of hormonal pathways - excessive estrogen signaling creates a hostile uterine environment for embryo development while simultaneously fueling oncogenic growth in mammary epithelium (Bartkowiak-Wieczorek et al., 2024). The resulting infertility often manifests as luteal phase defects, recurrent pregnancy loss, and endometriosis-associated subfertility, with treatment challenges due to the need to balance cancer risks with reproductive goals (Coccia and Nardone LRizzello, 2022).

## 5 Translational applications: from genetic screening to oncofertility

Clinical translation of these genetic links spans proactive (BRCA1 screening in POI) and reactive strategies (oncofertility preservation). This section prioritizes actionable protocols, such as PGT-M for TP53 and micro-TESE for BRCA2, to address dual risks.

### 5.1 Screening and counseling

Recent studies emphasize the importance of genetic evaluation in infertile patients, particularly those with a personal or strong family history of cancer (Wood and Rehman HtBedrosian, 2020). For women with Premature Ovarian Insufficiency, genetic counseling to discuss testing for *BRCA1/2* mutations could be considered, as these genes are implicated in ovarian aging through defective DNA repair (La and Mastellari, 2021). However, universal screening is not currently standard practice, as the penetrance of these genes for causing POI requires further epidemiological validation. The potential contribution of pathogenic variants in genes like BRCA, FMR1, or MCM9 to idiopathic POI highlights a link that warrants further investigation (Cimadomo et al., 2023). Additionally, in the context of a strong family history, Lynch syndrome (MLH1/MSH2 mutations) could be considered in the differential diagnosis for women with unexplained infertility, as these mutations can impair endometrial receptivity. When identified through family history, early genetic diagnosis allows for personalized fertility treatments, preimplantation genetic testing (PGT), and enhanced cancer surveillance (Zhao et al., 2022).

### 5.2 Oncofertility programs: fertility preservation before cancer therapy

Modern oncofertility programs prioritize egg/sperm cryopreservation before chemotherapy or radiation, significantly improving post-treatment reproductive outcomes (Pawłowski et al., 2023). Key advancements include: Oocyte cryopreservation: Dual ovarian stimulation (follicular + luteal phase) increases yield by 22% in cancer patients (Alvarez RRamanathan and Ramanathan, 2018). Sperm cryopreservation: Microfluidic sorting techniques now achieve 95% post-thaw viability (Kashaninejad and Shiddiky MJANguyen, 2018). Experimental methods: Ovarian tissue freezing shows promise, with 40% live birth rates in recent trials (Marco et al., 2024). Current ASCO guidelines (2024) mandate fertility counseling within 72 h of cancer diagnosis, as it reduces psychological distress and improves quality of life (Wadasadawala et al., 2024). Adolescents who preserve fertility before treatment have 3x lower depression rates during survivorship (Lehmann et al., 2024).

### 5.3 Assisted reproductive technologies (ART)

#### 5.3.1 PGT-M for high-risk variants (TP53)

**FIGURE 5:**
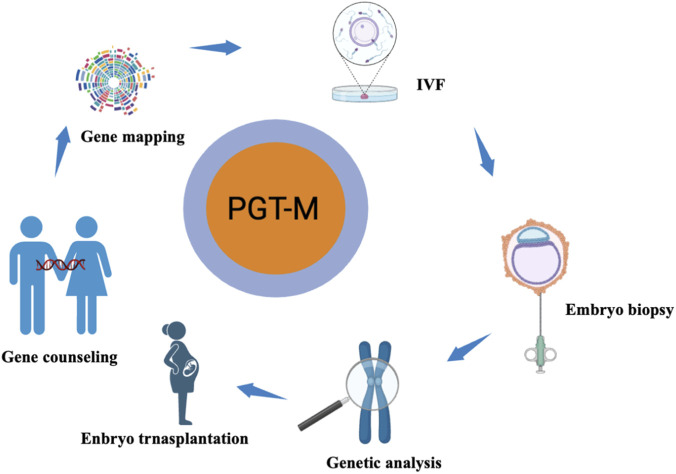
Key steps in IVF with genetic screening (PGT-M). This figure outlines the essential stages of *In Vitro* Fertilization (IVF) combined with Preimplantation Genetic Testing for Monogenic Disorders (PGT-M), from initial gene mapping and embryo biopsy to genetic analysis, counseling, and final embryo transfer. It highlights the integration of genetic screening to ensure healthy embryo selection before implantation. PGT-M provides a critical strategy for couples with hereditary cancer syndromes to reduce the risk of transmitting pathogenic variants to their offspring.

Recent advances in preimplantation genetic testing for monogenic disorders (PGT-M) now enable exclusion of embryos carrying pathogenic variants in cancer predisposition genes like TP53 (Albujja et al., 2024) (Figure 5). TP53-mutant embryos exhibit distinct metabolic profiles during early development, with altered glucose utilization detectable via time-lapse imaging (Wiecek, 2023). Clinical findings indicate substantial reductions in pediatric cancer risk when using PGT-M for families with Li-Fraumeni syndrome, though this approach continues to raise ethical questions about selecting against genetic conditions that manifest in adulthood (Calosci et al., 2023). Additional evidence demonstrates that combining PGT-M with mitochondrial DNA analysis can enhance assisted reproduction success rates, particularly for individuals carrying TP53mutations (Morales, 2024).

#### 5.3.2 ICSI for BRCA2-associated azoospermia

**FIGURE 6:**
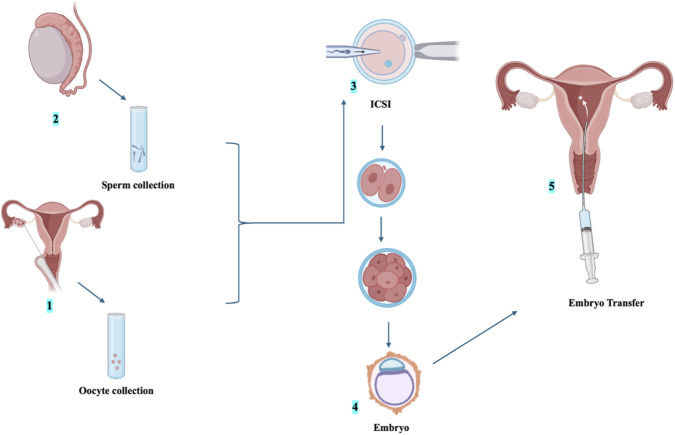
Overview of ICSI-assisted fertilization and embryo transfer. This figure illustrates the intracytoplasmic sperm injection (ICSI) process, starting with oocyte and sperm collection (steps 1–2), followed by ICSI fertilization and embryo development (steps 3–4), and ending with embryo transfer into the uterus (step 5) for potential implantation and pregnancy. ICSI is particularly valuable for severe male factor infertility, including cases of azoospermia where sperm is retrieved surgically, enabling biological parenthood where it was previously impossible.

*BRCA2’s* critical role in meiotic recombination creates unique challenges for male fertility preservation (Dias Nunes and Demeestere IDevos, 2023). Recent studies demonstrate that men with BRCA2-associated azoospermia often retain focal spermatogenesis, achieving 89% sperm retrieval rates via microdissection TESE (vs. 42% in idiopathic cases), making ICSI a viable option (Bayefsky et al., 2024) (Figure 6). However, concerns persist regarding potential transmission of *BRCA2* mutations and genomic instability in derived embryos, warranting mandatory PGT-M (Barrett et al., 2023).

## 6 Conclusion

The intricate relationship between infertility and cancer predisposition highlights fundamental The intricate relationship between infertility and cancer predisposition underscores fundamental connections between reproductive biology and oncogenesis, driven by shared molecular pathways in DNA repair, cell cycle regulation, and epigenetic maintenance. This review demonstrates that germline mutations in genes like BRCA1/2, TP53, and DNMT3A disrupt both reproductive function and cancer suppression, revealing a compelling biological link. Key clinical observations, such as the high sperm retrieval rates in BRCA2-associated azoospermia and the paradoxical, context-dependent actions of DNMT3A, highlight the complexity of these dual roles.

These findings make a strong case for further research into the utility of genetic screening in idiopathic infertility cases and advocate for integrated oncofertility approaches that combine PGT-M, fertility preservation, and enhanced cancer surveillance within a research context. Emerging strategies like antioxidant-supplemented ICSI for BRCA2 carriers and dual ovarian stimulation for fertility preservation demonstrate promising progress in addressing dual risks. Future longitudinal studies are critically needed to establish the true penetrance of infertility phenotypes in carriers of specific cancer-predisposing variants and to quantify the cancer risk in infertile populations with defined genetic etiologies.

However, significant challenges persist, including equitable access to these technologies and the ethical considerations surrounding embryo selection for adult-onset conditions. Future research should prioritize optimizing cryopreservation techniques, exploring therapies targeting shared pathways (PARP inhibitors for HR-deficient oocytes), and conducting long-term studies of ART outcomes in mutation carriers. By recognizing infertility as a potential biomarker for cancer risk and *vice versa*, we can advance personalized, multidisciplinary care models that bridge reproductive and oncologic medicine. This paradigm shift demands closer collaboration between reproductive endocrinologists, oncologists, and geneticists to translate these mechanistic insights into clinical practice, ultimately improving outcomes for affected individuals.
